# Overweight/obesity among health professionals and associated factors

**DOI:** 10.1590/0034-7167-2024-0254

**Published:** 2025-12-08

**Authors:** Sirlaine de Pinho, Lucineia de Pinho, Carla Silvana de Oliveira e Silva, Rene Ferreira da Silva, Victor Guilherme Pereira, Silvânia Paiva Santos, Maria Alice Moura Soares, Dulce Aparecida Barbosa

**Affiliations:** I Universidade Estadual de Montes Claros. Montes Claros, Minas Gerais, Brazil; II Instituto Federal de Educação, Ciência e Tecnologia do Sul de Minas Gerais. Machado, Minas Gerais, Brazil; III Universidade Federal de São Paulo. São Paulo, São Paulo, Brazil

**Keywords:** Obesity, Health Personnel, Risk Factors, Epidemiology, Public Health., Obesidad, Personal de Salud, Factores de Riesgo, Epidemiología, Salud Pública.

## Abstract

**Objectives:**

to estimate the prevalence of overweight/obesity and identify associated factors among health professionals.

**Methods:**

this cross-sectional study included professionals who had worked for at least six months in critical units of nine hospitals in northern Minas Gerais, Brazil. Sociodemographic, occupational, anthropometric, and health condition data were collected. Poisson regression with robust variance was applied to estimate prevalence ratios (PR) using SPSS 20.0.

**Results:**

a total of 490 health professionals participated, 65.9% were women, and 49.7% were aged 30-39 years. The prevalence of overweight/obesity was 61.8%. Associated factors included age ≥ 30 years, ≥ 5 years working in healthcare, elevated triglycerides, and elevated blood pressure.

**Conclusions:**

the prevalence of overweight/obesity among health professionals was high and associated with sociodemographic, occupational, and health factors, underscoring the need for support to promote lifestyle changes in this workforce.

## RESUMEN

**Objetivos:**

estimar la prevalencia de sobrepeso/obesidad e identificar los factores asociados en profesionales de la salud.

**Métodos:**

estudio transversal con trabajadores con al menos seis meses de antigüedad en áreas críticas de nueve hospitales del norte de Minas Gerais, Brasil. Se recopilaron datos sociodemográficos, ocupacionales, medidas antropométricas y condiciones de salud. El análisis utilizó el modelo de Poisson con varianza robusta para estimar razones de prevalencia, empleando el programa SPSS 20.0.

**Resultados:**

participaron 490 profesionales: 65,9% mujeres y 49,7% con edades entre 30 y 39 años. Se observó que el 61,8% presentaba sobrepeso u obesidad. Los factores asociados fueron edad ≥30 años, tiempo de trabajo en salud ≥5 años, triglicéridos y presión arterial alterados.

**Conclusiones:**

se identificó una elevada prevalencia de sobrepeso/obesidad entre profesionales de la salud, asociada a factores sociodemográficos, ocupacionales y de salud. Es necesario establecer apoyos que favorezcan cambios en el estilo de vida de estos trabajadores.

## INTRODUCTION

Over the past decades, studies worldwide have described obesity as an epidemic condition with a major public-health impact, ranking as the fifth leading cause of morbidity and mortality worldwide^(1,2)^. It places a substantial burden on healthcare spending because of its association with other chronic diseases, particularly cardiovascular disease, and has therefore become a priority for public policies^(1,2)^.

Obesity is considered a chronic, often inflammatory condition characterized by an abnormal or excessive accumulation of adipose tissue (fat). This accumulation results from a long-term energy imbalance between the daily calories consumed and expended^(2,3)^. In addition, the World Health Organization (WHO) defines obesity using the body mass index (BMI), the globally used metric for assessing overweight and obesity. BMI was calculated as BMI = weight (kg) / height (m^2^); adults with a BMI ≥ 30.0 were classified as obese according to WHO BMI cut-offs (overweight ≥ 25; obesity ≥ 30)^(2,4)^.

Obesity disrupts metabolic homeostasis and is a precursor to chronic conditions such as hypertension, type 2 diabetes mellitus (T2DM), and chronic kidney disease (CKD); it is also associated with an increased risk of neoplasms, dyslipidemia, and liver disease^(5,6)^. International studies have shown that people living with obesity and chronic conditions are more likely to develop cardiovascular and pulmonary complications; and during the COVID-19 pandemic, obesity was associated with greater odds of severe disease^(7-10)^.

Because obesity results from multifactorial interactions with heterogeneous pathogenesis, encompassing biological, behavioral, psychosocial, socioeconomic, and environmental factors, effective treatment is complex, making investment in prevention and early detection of risk factors even more important^(1,2,10)^. It is estimated that more than 650 million people worldwide are living with obesity, highlighting the enormous public-health burden^(3)^. In Brazil, in 2019, the prevalence of adult overweight and obesity was estimated at 61.7% and 26.8%, respectively^(11)^.

From this perspective, adverse working conditions, such as long working hours, excessive workloads, and prolonged exposure to stressful situations, commonly experienced by health professionals (particularly during the COVID-19 pandemic), may contribute to obesity among workers. These conditions also affect absenteeism and work productivity, with considerable consequences for institutions and society^(10,12,13)^. The work environment shapes dietary habits and physical activity, thereby affecting health and quality of life^(13)^.

## OBJECTIVES

To estimate the prevalence of overweight/obesity and identify factors associated with these conditions among health professionals.

## METHODS

### Ethical aspects

This study adhered to national and international ethical guidelines and obtained approval from the Research Ethics Committee of the State University of Montes Claros (CEP-Unimontes). The Informed Consent Form was signed by all participants in the study.

### Study design, period, and setting

This epidemiological, cross-sectional, analytical study used data from the survey titled *Compassion fatigue in health professionals: associated factors*. It was conducted with health professionals working in critical-care units (oncology, nephrology, neonatal intensive care, and emergency departments) of nine tertiary hospitals in the northern macroregion of Minas Gerais, Brazil. These hospitals provide care through the Brazilian Unified Health System (SUS) and are located in the following municipalities: Montes Claros (Hospital 1 - large; Hospital 2 - large; Hospital 3 - medium), Pirapora (Hospital 4 - medium), Janaúba (Hospital 5 - medium; Hospital 6 - small), Brasília de Minas (Hospital 7 - large), Salinas (Hospital 8 - medium), and Taiobeiras (Hospital 9 - medium).

The study followed the STROBE (Strengthening the Reporting of Observational Studies in Epidemiology) reporting guidelines^(14)^.

### Population, sample and selection criteria

The source population comprised 1,378 health professionals working in oncology, nephrology, neonatal intensive care, and emergency departments across the nine tertiary hospitals. Professional categories included nursing assistants/technicians, nurses, pharmacists, physiotherapists, physicians, nutritionists, and psychologists. Sample size was calculated assuming a 50% prevalence (to maximize sample size and given multiple outcomes), a 95% confidence level, 5.0% precision, and finite population correction; an additional 20% was added to account for possible nonresponse and losses, resulting in a minimum required sample size of 450 health professionals.

Simple random sampling was used: an exhaustive list of eligible professionals (N = 1,378) was compiled, and the sample was randomly selected using Excel for Windows^®^. The inclusion criterion was professionals with more than six months of work experience in the selected departments; the exclusion criteria were professionals on medical leave or on vacation at the time of data collection.

### Study protocol

Before data collection, authorization was obtained from each hospital. Health professionals were informed about the importance of participating in the study. Those who agreed, formalized by signing the Informed Consent Form (ICF), were enrolled. Data collection was carried out between January 2017 and April 2018. A self-administered questionnaire was used to gather sociodemographic, occupational, and physical-health information. Anthropometric measurements and venous blood samples for biochemical analysis were also obtained. The questionnaire was delivered to participants to be completed at home and included instructions on the return date and on the scheduling of laboratory and anthropometric examinations.

The investigated variables included sociodemographic characteristics (sex, age group, and marital status), occupational characteristics (work sector, years working in healthcare, role in the unit, and total weekly working hours), and health conditions (body mass index, triglycerides, total cholesterol, high-density lipoprotein [HDL] cholesterol, and blood pressure).

For anthropometric data collection, BMI was determined using a digital electronic scale (Wiso^®^ W721) and a stadiometer. Participants were weighed in their work clothes, standing with arms relaxed alongside their bodies. They were asked to remove their shoes, lab coats, jackets, watches, and other personal items. Height was measured using a portable stadiometer (range 35.0-213.0 cm; precision 0.1 cm). During measurement, participants were instructed to stand in the anatomical position, with their feet together and centered on the equipment, their head, buttocks, and heels against the wall, and their face directed forward in the Frankfort plane^(15)^. The stadiometer bar was lowered to the top of the head, and the reading was taken after a normal expiration. For BMI classification, the WHO criteria were adopted: underweight, < 18.5 kg/m^2^; normal weight, 18.5-24.9 kg/m^2^; overweight, 25.0-29.9 kg/m^2^; and obesity, ≥ 30.0 kg/m^2(16)^.

Biochemical variables (total cholesterol, HDL-cholesterol, and triglycerides) were measured from a single venous blood sample collected from the antecubital vein after 12 hours of fasting and analyzed by the enzymatic colorimetric method (LabmaxPlenno^®^). Reference values followed the Brazilian Guideline on Dyslipidemias and Atherosclerosis Prevention^(17)^, classified as “normal” e “altered”, respectively: total cholesterol (< 190 mg/dL; ≥ 190 mg/dL), HDL-cholesterol (> 40 mg/dL; ≤ 40 mg/dL), and triglycerides (< 150 mg/dL; ≥ 150 mg/dL).

Blood pressure was measured according to the 7th Brazilian Guideline for Arterial Hypertension^(18)^, using a validated automatic wrist blood pressure monitor (Omron^®^ HEM-6123). Three measurements were taken on each occasion: at the initial contact during questionnaire delivery and again during anthropometric assessment. For analysis, the mean of the last two readings was considered, with the first discarded. Blood pressure was classified as “normal” for systolic blood pressure (SBP) ≤ 120 mmHg and diastolic blood pressure (DBP) ≤ 80 mmHg; “prehypertension” for SBP 121-139 mmHg or DBP 81-89 mmHg; and “hypertension” for SBP ≥ 140 mmHg or DBP ≥ 90 mmHg. Participants were also classified as hypertensive if they reported regular use of antihypertensive medication, regardless of measured values. When SBP and DBP fell into different categories, the higher value was used. For analysis, blood pressure was dichotomized into “normal” and “altered” categories (prehypertension and hypertension).

### Analysis of results and statistics

The outcome variable, overweight/obesity, was dichotomized into “no” (BMI <25 kg/m^2^) and “yes” (BMI ≥ 25 kg/m^2^). The independent variables comprised sociodemographic, occupational, and physical-health characteristics.

Descriptive analyses of all variables were first performed using absolute and relative frequencies. The prevalence of overweight/obesity was estimated with 95% confidence intervals (95% CI). Bivariate analyses were then conducted, followed by multivariable analyses, using Poisson regression with robust variance. Variables with p ≤0.20 in the bivariate analysis were selected for inclusion in the multivariable analysis.

Multivariable analysis employed a hierarchical Poisson regression model with robust variance, adapted from Siqueira et al.^(13)^. The model was structured into three levels of variables: distal (sex, age group, marital status, and having children), intermediate (work sector, years working in healthcare, work shift, role in the unit, and total weekly working hours), and proximal (leave of absence, triglycerides, total cholesterol, HDL-cholesterol, blood pressure, stress, and depression). “Sex” and “age” were considered a priori as confounders and were retained in the adjusted model.

The modeling process began with the inclusion of distal variables. Those with p <0.05 were carried forward to the next block, which included intermediate variables. The same procedure was applied to proximal variables. The final model was adjusted for work sector and role in the unit. Only variables with *p* < 0.05 in the final hierarchical model were presented. Model fit was evaluated using the deviance test. All analyses were conducted using the Statistical Package for the Social Sciences (SPSS), version 20.0.

## RESULTS

A total of 490 health professionals participated in the study, of whom 61.8% (95% CI: 57.5-66.1) were classified as overweight/obese. The sample was predominantly female (65.9%), aged 30-39 years (49.7%), and with a total weekly working hours of up to 44 hours per week (56.5%). Among participants, 14.1% (n = 66) worked in the ICU, 31.6% (n = 148) in nephrology, 22.8% (n = 107) in oncology, and 31.6% (n = 148) in the emergency department. By role, 66.3% (n = 311) were nursing assistants/technicians, 15.6% (n = 73) nurses, 8.7% (n = 41) physicians, and 9.4% (n = 44) other professionals. Overall, 44.3% (n = 217) of participants had altered total cholesterol, 50.3% (n = 246) altered HDL-cholesterol, 28.8% (n = 141) altered triglycerides, and 36.1% (n = 177) altered blood pressure levels.

Table 1 presents the results of the bivariate and multivariable analyses, with estimates of crude and adjusted prevalence ratios for overweight/obesity. After model adjustment, regardless of work sector and role in the unit, the variables associated with overweight/obesity were: Distal level - age group (30-39 years, p = 0.018; ≥ 40 years, p < 0.001); Intermediate level - years working in healthcare (5-10 years, p = 0.018; 11-15 years, p = 0.007; >15 years, p = 0.034); and Proximal level - altered triglycerides (p = 0.006) and altered blood pressure (p < 0.001).

**Table 1:** Hierarchical multiple model with crude and adjusted prevalence ratios for overweight/obesity according to sociodemographic, occupational, and health characteristics among health professionals, Northern macroregion of Minas Gerais, Brazil, 2017-2018

Variables	_crude_PR (95% CI)		P	_crude_PR (95% CI)	P
**Distal level (Sociodemographic)**					
**Sex**					
Male	1.00		-	1.00	-
Female	0.89 (0.77-1.02)		0.089	0.86(0.77-1.02)	0.086
**Age group**					
20-29 years	1.00		-	1.00	-
30-39 years	1.29 (1.05-1.59)		0.016	1.29 (1.05-1.58)	0.018^*^
≥ 40 years	1.47 (1.19-1.83)		0.000	1.47 (1.19-1.82)	<0.001^*^
**Marital status**					
Without partner	1.00		-	-	
With partner	1.14 (0.98-1.32)		0.086	-	-
		**Block 1:** *Deviance:* 282.938; gl=482; p>0.900			
**Intermediate level (Occupational)**					
**Years working in healthcare**					
≤ 5 years	1.00		-	1.00	
5-10 years	1.44 (1.11-1.88)		0.006	1.40 (1.06-1.85)	0.018^*^
11-15 years	1.60 (1.24-2.08)		0.000	1.53 (1.13-2.07)	0.007^*^
15 years	1.65 (1.28-2.15)		0.000	1.42 (1.03-1.97)	0.034^*^
**Total weekly working hours**					
≤ 44 h/week	1.00		-	-	
45-60 h/week	0.90 (0.75-1.08)		0.240	-	-
≥ 61 h/week	1.21 (1.02-1.43)		0.029	-	-
		**Block 2:** *Deviance:* 264.492; gl=451; p>0.900			
**Proximal level (Health conditions)**					
**Total cholesterol**					
Normal	1.00		-	-	
Altered	1.11(0.97-1.28)		0.143	-	-
**HDL-cholesterol**					
Normal	1.00		-	-	
Altered	0.88 (0.76-1.01)		0.074	-	-
**Triglycerides**					
Normal	1.00		-	1.00	
Altered	1.25 (1.09-1.43)		0.002	1.21 (1.06-1.40)	0.006^*^
**Blood pressure**					
Normal	1.00			1.00	
Altered	1.46 (1.28-1.66)		0.000	1.34 (1.15-1.55)	<0.001^*^
		**Block 3:** *Deviance:* 256.296; gl=448; p>0.900			

PR - prevalence ratio; CI - confidence interval;

* significant at the 0.05 level;

** model adjusted for “work sector” and “role in the unit” variables.

## DISCUSSION

This study demonstrated that more than half of health professionals were classified as overweight/obese, an outcome associated with age group, blood pressure, triglycerides, and years working in healthcare. Overweight/obesity among health professionals is a relevant issue, as it compromises quality of life, increases morbidity in this population, and may reduce life expectancy. Individuals with obesity are more likely to develop noncommunicable diseases (NCDs), such as type 2 diabetes mellitus, malignant neoplasms, coronary heart disease, and stroke^(19,20)^.

The high prevalence observed in this study is similar to that reported in a survey of 4,489 government-employed health professionals working in health clinics and hospitals across 11 districts on the east coast of Peninsular Malaysia: 33.1% of individuals were overweight and 21.1% were obese^(21)^. Similarly, a study conducted in England estimated obesity prevalence among health professionals and found high rates among nurses (25.1%), unregistered care workers (31.9%), and other health professionals (14.4%)^(22)^. In addition, a study involving Family Health Strategy (FHS) teams participating in the Health-Promoting Units program in Montes Claros, northern Minas Gerais, found that 53.5% of professionals were overweight^(13)^.

The prevalence of overweight/obesity among health professionals was similar to that observed in the general adult population: 61.8% among health professionals and 61.4% in the general adult Brazilian population. In a study conducted between 2017 and 2018 in 12 European countries (Bulgaria, England, France, Germany, Greece, Ireland, Italy, Latvia, Poland, Portugal, Romania, and Spain), involving 10,810 participants, 48.1% were overweight and 12.6% were obese^(23)^. By contrast, an investigation in Nigeria found that 25.0% of individuals were overweight, and 14.3% were obese^(24)^.

The prevalence of overweight/obesity among health professionals may be linked to factors such as irregular and long working hours, unhealthy dietary habits, and high stress levels in the workplace, conditions frequently present in this profession. Health professionals are exposed to demanding working conditions, such as heavy workloads, irregular schedules, and extended shifts; which compromise their quality of life and predispose them to unhealthy behaviors, including poor diet and physical inactivity, thereby increasing the risk of overweight and obesity^(13,21)^.

According to recent evidence, health literacy is regarded as essential for controlling overweight/obesity and fostering the awareness needed to address these condition. The role of health professionals in educating the population is therefore highly relevant^(25)^. In this context, overweight/obesity among health professionals may undermine their credibility with patients, reducing adherence to the guidance provided. Moreover, professionals with obesity often feel less confident about counseling patients on healthy eating and the importance of physical activity^(21)^.

In the present study, a higher prevalence of overweight/obesity was observed among health professionals aged 30-39 years. These findings are consistent with those of a population-based cohort study in the United Kingdom, which showed that the association between BMI and mortality was stronger in adults younger than 50 years^(26)^. Similarly, a study conducted in Recife, the capital of the state of Pernambuco, also found a statistically significant association between overweight/obesity and age group, with a higher prevalence among adults aged 30-39 years, who accounted for 27.5% of those classified as obese^(27)^. Decreased physical activity and age-related physiological changes contribute to increased body fat stores, exceeding acceptable anthropometric standards^(27)^.

Our study showed that overweight/obesity was associated with altered blood pressure and triglycerides. The fat accumulation that occurs in obesity leads to dyslipidemia, characterized by elevated levels of total cholesterol (TC), low-density lipoprotein (LDL) cholesterol, and triglycerides^(28)^. Overweight/obesity increases adipokine production, leading to a chronic inflammatory state, which contributes to metabolic complications such as hypertension^(29)^. When overweight/obesity and hypertension coexist, adverse cardiac remodeling occurs, increasing the risk of heart failure^(30)^. In a study conducted in northeastern Minas Gerais, Brazil, with individuals aged 18-88 years, elevated triglyceride levels were identified as a significant risk factor for cardiovascular diseases and, consequently, hypertension^(31)^. Similarly, in an investigation conducted in the Kailuan community in China, both higher triglyceride levels and greater variability in triglyceride indices were independently associated with an increased incidence of cardiovascular diseases^(32)^.

In this study, overweight/obesity was also associated with longer time working in healthcare. A study conducted with health workers in Rio de Janeiro found that 53.5% were overweight (36.7% overweight and 16.8% obese). This finding is consistent with the Malaysian study mentioned earlier, in which 33.1% of health professionals were overweight and 21.1% were obese. Evidence suggests that the longer health professionals are exposed to the exhausting routines inherent in healthcare, the more their lifestyle habits become compromised, leading to irregular and prolonged working hours and unhealthy dietary practices, which predispose them to obesity^(13,21)^.

### Study limitations

This cross-sectional study has some limitations. Because it provides only a snapshot of exposure and outcome, it does not allow for assessing the temporal relationship between cause and effect. In addition, selection bias and response bias may have affected data collection and, consequently, the accuracy of the results. Furthermore, although the blood pressure monitor used was validated, it may be less precise than devices recommended for epidemiological studies.

### Contributions to the field

As the global prevalence of overweight/obesity among health professionals has risen rapidly and continuously in recent decades, there is an urgent need to implement strategies, such as adjusting irregular shifts and long working hours, to support healthier routines and improve these workers’ quality of life. This study is relevant as it identifies the current and potential consequences for health professionals. Physical and psychological impacts lead to reduced quality of life and reduced capacity to work, thereby increasing the incidence of illness.

## CONCLUSIONS

The high prevalence of overweight/obesity among health professionals was associated with sociodemographic, occupational, and health-related characteristics, including aging, career duration, and health indicators.

These findings underscore the importance of implementing programs that encourage regular physical activity and healthy dietary habits to improve quality of life and prevent overweight/obesity-related diseases. It is also essential to establish support networks that provide continuous assistance to facilitate behavior change. Such initiatives should be complemented by occupational health policies and targeted interventions aimed at promoting a healthier and more balanced work environment. Finally, longitudinal studies with samples from different hospital settings are recommended.

## Data Availability

The research data are available only upon request.
