# Erratum: Overweight/obesity among health professionals and associated factors

**DOI:** 10.1590/0034-7167.202679e0220240254

**Published:** 2026-08-07

In the article “Overweight/obesity among health professionals and associated factors”, with DOI number: https://doi.org/10.1590/0034-7167-2024-0254, published in Revista Brasileira de Enfermagem, 2025;78(6):e20240254:

Included:

**FUNDING**

*Fundação de Amparo à Pesquisa do Estado de Minas Gerais* (Fapemig) - Process APQ-04716-24, approved by Public Notice No. 009/2024 - Strengthening and consolidation of research at UEMG and UNIMONTES.

Prof. PhD Dulce Barbosa

*Editor in Chief*

