# Supplementary material for: Overweight/obesity among health professionals and associated factors
Source: Rev Bras Enferm. 2025 Dec 8;78(6):e20240254. doi: 10.1590/0034-7167-2024-0254 (PMC12707615; doi:10.1590/0034-7167-2024-0254)
Supplement: Translation

# Translation: Sobrepeso/obesidade em profissionais de saúde e fatores associados

**DOI:** 10.1590/0034-7167-2024-0254pt

**Authors:** Sirlaine de Pinho, Lucineia de Pinho, Carla Silvana de Oliveira e Silva, Rene Ferreira da Silva, Victor Guilherme Pereira, Silvânia Paiva Santos, Maria Alice Moura Soares, Dulce Aparecida Barbosa

**Affiliations:** I Universidade Estadual de Montes Claros. Montes Claros, Minas Gerais, Brasil; II Instituto Federal de Educação, Ciência e Tecnologia do Sul de Minas Gerais. Machado, Minas Gerais, Brasil; III Universidade Federal de São Paulo. São Paulo, São Paulo, Brasil

**Keywords:** Obesidade, Pessoal de Saúde, Fatores de Risco, Epidemiologia, Saúde Pública.

## Abstract

**Objetivos:**

estimar a prevalência de sobrepeso/obesidade e identificar fatores associados em profissionais de saúde.

**Métodos:**

estudo transversal com trabalhadores atuantes há pelo menos seis meses em áreas críticas de nove hospitais no norte de Minas Gerais. Coletaram-se informações sociodemográficas, ocupacionais, medidas antropométricas e condições de saúde. A análise utilizou o modelo de Poisson com variância robusta para estimar razões de prevalência, no SPSS 20.0.

**Resultados:**

participaram 490 profissionais: 65,9%, mulheres; e 49,7%, com idade entre 30 e 39 anos. Verificou-se que 61,8% apresentavam sobrepeso ou obesidade. Os fatores associados foram idade ≥30 anos, tempo de trabalho na saúde ≥5 anos, triglicérides e pressão arterial alterados.

**Conclusões:**

há elevada prevalência de sobrepeso/obesidade entre profissionais de saúde, associada a fatores sociodemográficos, ocupacionais e de saúde, sendo necessário oferecer suporte para promover mudanças no estilo de vida desses trabalhadores.

## INTRODUÇÃO

Nas últimas décadas, estudos pelo mundo têm considerado a obesidade como uma condição epidêmica de significativo impacto na saúde pública, sendo classificada como a quinta principal causa de morbimortalidade global. Ela exerce forte pressão sobre os gastos dos serviços de saúde devido à sua associação com o desenvolvimento de outras doenças crônicas, sobretudo as cardiovasculares, tornando-se, portanto, uma prioridade nas políticas públicas^(1,2)^.

Nessa direção, a obesidade é conceituada como uma condição inflamatória, muitas vezes crônica, causada pelo acúmulo anormal ou excessivo de tecido adiposo (gordura), que resulta do desequilíbrio energético longitudinal entre as calorias consumidas e as calorias necessárias e gastas ao longo do dia^(2,3)^. Adicionalmente, a Organização Mundial de Saúde (OMS) define a obesidade por meio da aplicação do Índice de Massa Corporal (IMC), que é a unidade de medida mundialmente destinada para verificar sobrepeso e obesidade, calculado pela fórmula: IMC = peso (kg) / [altura (m)]^2^. Considera “obeso” quando um indivíduo apresenta o IMC ≥ a 30,0^(2,4)^.

A obesidade afeta a homeostase metabólica, sendo precursora de doenças crônicas como a hipertensão arterial sistêmica (HAS), diabetes *mellitus* (DM) tipo 2 e doença renal crônica (DRC), favorecendo também o desenvolvimento de neoplasias, hiperlipidemia e hepatopatias^(5,6)^. Estudos internacionais constataram que indivíduos obesos e com doenças crônicas são mais propensos a desenvolver complicações cardiovasculares e pulmonares^(7,8)^; e, no contexto da pandemia de COVID 19, observou-se maior probabilidade de manifestar formas graves dessa doença^(9,10)^.

Por resultar da interação multifatorial, com patogênese heterogênea relacionada a fatores biológicos, comportamentais, psicossociais, socioeconômicos e ambientais, torna-se complexo alcançar resultados eficazes no tratamento da obesidade, fazendo que os investimentos na sua prevenção e detecção de fatores de risco sejam ainda mais relevantes^(1,2,10)^. Estima-se que mais de 650 milhões de pessoas no mundo a tenham desenvolvido nas últimas três décadas, um número que representa a dimensão avassaladora desse processo mórbido na saúde pública mundial^(3)^. No Brasil, em 2019, a taxa de prevalência de pessoas adultas com sobrepeso e obesidade foi estimada em 61,7 % e 26,8%, respectivamente^(11)^.

Dessa perspectiva, destaca-se que condições adversas no contexto laboral, como jornadas longas de trabalho, demandas excessivas e exposição longitudinal a situações estressantes, frequentemente vivenciadas pelos profissionais da saúde (especialmente durante a pandemia da COVID-19), podem contribuir para o surgimento da obesidade na população trabalhadora. Essas condições também impactam as taxas de absenteísmo e produtividade no trabalho, com consequências consideráveis para as instituições e para a sociedade^(10,12,13)^. Cabe salientar que o cenário de trabalho interfere no padrão de vida, nos hábitos alimentares e na prática de exercícios físicos do indivíduo, refletindo-se na sua condição de saúde e qualidade de vida^(13)^.

## OBJETIVOS

Estimar a prevalência de sobrepeso/obesidade e identificar os fatores associados em profissionais de saúde.

## MÉTODOS

### Aspectos éticos

O estudo aderiu às diretrizes de ética nacionais e internacionais e obteve aprovação do Comitê de Ética em Pesquisa da Universidade Estadual de Montes Claros (CEP-Unimontes). O Termo de Consentimento Livre e Esclarecido foi assinado por todos os indivíduos envolvidos no estudo.

### Desenho, período e local do estudo

Este estudo utilizou dados da pesquisa intitulada “Fadiga por Compaixão em Profissionais da saúde: fatores associados”. Trata-se de um estudo epidemiológico, transversal e analítico. Foi conduzido com profissionais de saúde atuantes em setores que atendem pacientes críticos (oncologia, nefrologia, terapia intensiva neonatal e pronto-socorro) em nove hospitais de alta complexidade da macrorregião do norte de Minas Gerais, Brasil. Esses hospitais atendem pelo Sistema Único de Saúde e estão localizados nos municípios de Montes Claros (Hospital 1: grande porte; Hospital 2: grande porte 2; Hospital 3: médio porte), Pirapora (Hospital 4: médio porte), Janaúba (Hospital 5: médio porte, Hospital 6: pequeno porte), Brasília de Minas (Hospital 7: grande porte), Salinas (Hospital 8: médio porte) e Taiobeiras (Hospital 9: médio porte).

O estudo seguiu as diretrizes do referencial STROBE (*Strengthening the Reporting of OBservational studies in Epidemiology*)^(14)^.

### População, amostra e critérios de seleção

A população do estudo foi constituída por 1.378 profissionais de saúde atuantes em setores de oncologia, nefrologia, terapia intensiva neonatal e pronto-socorro dos nove hospitais de alta complexidade da macrorregião norte de Minas Gerais. Foi composta por auxiliares/técnicos de enfermagem, enfermeiros, farmacêuticos, fisioterapeutas, médicos, nutricionistas e psicólogos. O tamanho da amostra foi calculado assumindo uma prevalência de 50% (para maximizar o tamanho amostral e devido aos diversos desfechos), intervalo de 95% de confiança (IC 95%), nível de precisão de 5,0% e correção para população finita. Estabeleceu-se um acréscimo de 20% para compensar as possíveis não respostas e perdas, resultando no número mínimo de 450 profissionais de saúde.

Na seleção da amostra, adotou-se a técnica de amostragem aleatória simples. Para tal, foi elaborada uma lista de todos os profissionais elegíveis (N = 1.378). Em seguida, por meio do Excel for Windows^®^, foi realizado o sorteio aleatório da amostra de profissionais a serem. Foram incluídos os profissionais de saúde com mais de seis meses de atuação nos referidos setores e excluídos aqueles em licença médica ou em período de férias no momento da coleta de dados.

### Protocolo do estudo

Antes de iniciar a coleta de dados, obteve-se a autorização de cada hospital. Em seguida, os profissionais foram sensibilizados sobre a importância de sua participação na pesquisa. Com a concordância de cada um, formalizada por meio da assinatura do Termo de Consentimento Livre e Esclarecido, iniciou-se a coleta dos dados, que ocorreu entre janeiro de 2017 e abril de 2018. Foi utilizado um questionário autoaplicável composto por questões referentes às características sociodemográficas, ocupacionais e de saúde física dos participantes. Também foi feita a aferição dos dados antropométricos e a coleta de sangue para análise bioquímica. O questionário foi entregue aos profissionais para ser preenchido em domicílio: continha informações sobre a data de devolução e realização dos exames laboratoriais e antropométricos.

As variáveis investigadas foram: características sociodemográficas (sexo, faixa etária e estado civil), ocupacionais (setor de trabalho, tempo de trabalho na área de saúde, função no setor e carga horária total de trabalho) e condições de saúde (Índice de Massa Corporal, triglicérides, colesterol total, colesterol da lipoproteína de alta densidade e pressão arterial).

No procedimento de coleta de dados antropométricos, o IMC foi determinado com uso de balança eletrônica digital (marca Wiso^®^, modelo W721) e de um estadiômetro. Os profissionais foram pesados com a roupa de trabalho, tendo os braços relaxados ao longo do corpo. Foi solicitada a retirada de calçados, jalecos, casacos, relógios e outros objetos. A estatura foi avaliada por meio de um estadiômetro portátil, com escala de 35,0 a 213,0 cm e precisão de 0,1 cm. Durante a mensuração, foram orientados a manterem-se em posição anatômica, com os pés juntos e centralizados no equipamento, encostando cabeça, nádegas e calcanhares na parede e tendo a face voltada para frente em Plano de Frankfurt^(15)^. A régua do estadiômetro foi deslocada até a cabeça do participante, sendo realizada a leitura após uma expiração normal. Para a classificação em função do IMC, foram adotados os critérios da OMS: baixo peso, < 18,5 kg/m^2^; eutrófico, 18,5 a 24,9 kg/m^2^; sobrepeso, 25 a 29,9 kg/m^2^; e obeso, ≥ 30 kg/m^2(16)^.

As variáveis bioquímicas (colesterol, HDL-colesterol e triglicérides) foram coletadas em amostra única de sangue venoso antecubital após 12 horas de jejum, mediante análise laboratorial utilizando o método colorimétrico enzimático (aparelho LabmaxPlenno^®^). Foram adotados os valores de referência recomendados pela Diretriz Brasileira de Dislipidemias e Prevenção da Aterosclerose^(17)^, em que foram classificados como “normal” e “alterado”, respectivamente: colesterol (< 190 mg/dl e ≥ 190 mg/dl), HDL-colesterol (> 40 mg/dl e ≤ 40 mg/dl) e triglicérides (< 150 mg/dl e ≥ 150 mg/dl).

Para a coleta de dados da pressão arterial, seguiram-se as orientações da 7ª Diretriz Brasileira de Hipertensão Arterial, usando o Monitor de Pressão Arterial Automático de Pulso Control (HEM-6123) - Omron^®^ (validado). Foram realizadas três aferições em cada seguinte ocasião: abordagem inicial ao profissional e entrega dos questionários; e coleta dos dados antropométricos. Para análise estatística, considerou-se a média das duas últimas aferições, descartando-se a primeira. Os níveis pressóricos dos participantes foram descritos como “normal” para pressão arterial sistólica (PAS) com valor ≤ 120 mmHg e pressão arterial diastólica (PAD) com valor ≤ 80 mmHg; como “pré-hipertensão” para valores médios de PAS entre 121 e 139 mmHg e de PAD entre 81 e 89 mmHg; e como “hipertensão” para valores médios de PAS ≥ 140 mmHg e de PAD ≥ 90 mmHg. Os participantes eram também classificados como “hipertensos” se relatassem uso periódico de medicamento anti-hipertensivo independentemente do valor de pressão arterial aferido. Quando a PAS e a PAD enquadravam-se em categorias diferentes, a maior medida foi considerada para classificação da pressão arterial^(18)^. A variável “pressão arterial” foi categorizada em “normal” e “alterada” (pré-hipertensão e hipertensão).

### Análise dos resultados e estatística

A variável “sobrepeso/obesidade”, dicotomizada em “não” (IMC < 25 kg/m^2^) e “sim” (IMC ≥ 25 kg/m^2^), foi considerada variável-desfecho do estudo, ao passo que as características sociodemográficas, ocupacionais e condições de saúde física foram estabelecidas como variáveis independentes.

Inicialmente, as análises descritivas de todas as variáveis foram feitas mediante frequências simples e relativa. Estimou-se a prevalência de sobrepeso/obesidade com intervalo de 95% de confiança. Em seguida, foram realizadas as análises bivariada e múltipla entre a variável dependente e as variáveis independentes, adotando-se o modelo de regressão de Poisson, com variância robusta. As variáveis que apresentaram *p* ≤ 0,20 na etapa bivariada foram selecionadas para análise múltipla.

Na análise múltipla, utilizou-se o modelo de regressão de Poisson, hierarquizado e com variância robusta, adaptado de Siqueira et al.^(13)^ O modelo foi estruturado em três níveis de variáveis: distal (sexo, faixa etária, estado civil e tem filhos), intermediário (setor de trabalho, tempo de trabalho na área de saúde, turno, função no setor e carga horária total de trabalho) e proximal (tirou licença, triglicérides, colesterol total, colesterol da lipoproteína de alta densidade, pressão arterial, estresse e depressão). A priori, “sexo” e “idade” foram consideradas variáveis de confusão, sendo mantidas no modelo ajustado.

A modelagem hierarquizada iniciou-se com a inclusão das variáveis do nível distal. Aquelas com *p* < 0,05 foram inseridas no modelo subsequente, o qual incluiu as variáveis do nível intermediário. Por fim, seguiu-se o mesmo processo para as variáveis proximais. O modelo final foi ajustado pelas variáveis “setor de trabalho” e “função no setor”. Nos resultados, constaram apenas as variáveis com *p* < 0,05 no modelo final hierarquizado. Para avaliar a qualidade do ajuste do modelo, foi utilizado o teste de *deviance.* Todas as análises foram feitas com uso do pacote computadorizado *Statistical Package for the Social Sciences* (SPSS), versão 20.0.

## RESULTADOS

Participaram do estudo 490 trabalhadores da área da saúde, dos quais 61,8%, IC95% [57,5% - 66,10%], apresentaram sobrepeso/obesidade. A amostra foi constituída majoritariamente por profissionais do sexo feminino (65,9%), com faixa etária de 30 a 39 anos (49,7%), com carga horária de até 44 h/semanais (56,5%). Entre os profissionais, 14,1% (n = 66) atuavam no CTI, 31,6% (n = 148) na nefrologia, 22,8% (n = 107) na oncologia e 31,6% (n = 148) no pronto-socorro. A função no setor era de auxiliar/técnico em enfermagem (66,3%, n = 311), enfermeiro (15,6%, n = 73), médico (8,7%, n = 41) e outras (9,4%, n = 44). Os resultados revelaram que 44,3% (n = 217) dos participantes apresentaram alterações nos níveis de colesterol total, 50,3% (n = 246) no colesterol HDL, 28,8% (n = 141) nos triglicérides e 36,1% (n = 177) na pressão arterial.

A Tabela 1 apresenta os resultados da análise bivariada e múltipla, com estimativas das razões de prevalência bruta e ajustada para a variável “sobrepeso/obesidade”. Após o ajuste do modelo, independentemente do setor de trabalho e da função no setor, as variáveis associadas ao sobrepeso/obesidade foram: Nível distal - faixa etária (30 a 39 anos, p = 0,018; 40 anos ou mais, p < 0,001); Nível intermediário - tempo de trabalho na área da saúde (entre 5 e 10 anos, p = 0,018; entre 10 e 15 anos, p = 0,007; acima de 15 anos, p = 0,034); e Nível proximal - triglicérides alterados (p = 0,006) e pressão arterial alterada (< 0,001).

**Tabela 1:** Modelo múltiplo hierarquizado, com razão de prevalência (RP) bruta e ajustada para a variável “sobrepeso/obesidade” segundo características sociodemográficas, ocupacionais e condições de saúde entre trabalhadores da saúde, Macrorregião norte de Minas Gerais, Brasil, 2017-2018

Variáveis	RP_bruta_ (IC 95%)		P	RP_ajustada_ (IC 95%)	P
**Nível Distal (Sociodemográficas)**					
**Sexo**					
Masculino	1,00		-	1,00	-
Feminino	0,89 (0,77-1,02)		0,089	0,86(0,77-1,02)	0,086
**Faixa etária**					
20 a 29 anos	1,00		-	1,00	-
30 a 39 anos	1,29 (1,05-1,59)		0,016	1,29 (1,05-1,58)	0,018^*^
40 anos ou mais	1,47 (1,19-1,83)		0,000	1,47 (1,19-1,82)	<0,001^*^
**Estado civil**					
Sem companheiro	1,00		-	-	
Com companheiro	1,14 (0,98-1,32)		0,086	-	-
		**Bloco 1:** *Deviance:* 282,938; gl=482; p>0,900			
Nível intermediário (Ocupacionais)^**^					
**Tempo de trabalho na área da saúde**					
Até 5 anos	1,00		-	1,00	
Entre 5 e 10 anos	1,44 (1,11-1,88)		0,006	1,40 (1,06-1,85)	0,018^*^
Entre 11 e 15 anos	1,60 (1,24-2,08)		0,000	1,53 (1,13-2,07)	0,007^*^
Acima de 15 anos	1,65 (1,28-2,15)		0,000	1,42 (1,03-1,97)	0,034^*^
**Carga horária total trabalho**					
Até 44 h/semana	1,00		-	-	
Entre 45 h e 60 h	0,90 (0,75-1,08)		0,240	-	-
61 h ou mais	1,21 (1,02-1,43)		0,029	-	-
		**Bloco 2:** *Deviance:* 264,492; gl=451; p>0,900			
Nível proximal (Condições de saúde)^**^					
**Colesterol Total**					
Normal	1,00		-	-	
Alterado	1,11(0,97-1,28)		0,143	-	-
**Colesterol HDL**					
Normal	1,00		-	-	
Alterado	0,88 (0,76-1,01)		0,074	-	-
**Triglicérides**					
Normal	1,00		-	1,00	
Alterado	1,25 (1,09-1,43)		0,002	1,21 (1,06-1,40)	0,006^*^
**Pressão arterial**					
Normal	1,00			1,00	
Alterada	1,46 (1,28-1,66)		0,000	1,34 (1,15-1,55)	<0,001^*^
		**Bloco 3:** *Deviance:* 256,296; gl=448; p>0,900			

RP - razão de prevalência, IC - intervalo de confiança,

* significante ao nível de 0,05;

** modelo ajustado pelas variáveis “setor de trabalho” e “função no setor”.

## DISCUSSÃO

Este estudo demonstrou que mais da metade dos profissionais apresentaram sobrepeso/obesidade - desfecho que teve associação com a faixa etária, pressão arterial, triglicérides e tempo de trabalho na área. O sobrepeso/obesidade entre os profissionais de saúde é uma questão relevante, visto que compromete a qualidade de vida e aumenta a morbidade nessa população. Além disso, suas complicações podem reduzir a expectativa de vida. Os indivíduos obesos são mais propensos a doenças não transmissíveis (DCNTs), como diabetes tipo 2, doenças malignas, doença cardíaca coronária e acidente vascular cerebral^(19,20)^.

A elevada prevalência do desfecho que encontramos assemelha-se aos resultados de um estudo feito com 4.489 profissionais de saúde do governo, abrangendo todas as clínicas de saúde e hospitais em 11 distritos da região da costa leste da Península da Malásia: 33,1% dos indivíduos estavam acima do peso, e 21,1% eram obesos^(21)^. Já em um estudo feito na Inglaterra, em que se estimou a prevalência de obesidade entre profissionais de saúde, encontrou-se um nível alto entre enfermeiros (25,1%), cuidadores não registrados (31,9%) e outros profissionais de saúde (14,4%)^(22)^. Ademais, em uma pesquisa com profissionais das equipes da Estratégia Saúde da Família (ESF), participantes do programa Unidades Promotoras da Saúde, no município de Montes Claros, norte de Minas Gerais, 53,5% dos profissionais tinham excesso de peso^(13)^.

Quando comparada a prevalência de sobrepeso/obesidade em profissionais de saúde com a da população adulta geral, observam-se valores semelhantes: 61,8% entre os profissionais de saúde e 61,4% na população adulta brasileira. Em um estudo realizado entre 2017 e 2018 em 12 países europeus (Bulgária, Inglaterra, França, Alemanha, Grécia, Irlanda, Itália, Letônia, Polônia, Portugal, Romênia e Espanha), com 10.810 participantes, 48,1% apresentavam sobrepeso, e 12,6% eram obesos^(23)^. Já em uma investigação realizada na Nigéria, 25,0% indivíduos tinham sobrepeso, e 14,3% eram obesos^(24)^.

A prevalência de sobrepeso/obesidade entre profissionais de saúde pode estar associada a fatores como horários de trabalho irregulares e prolongados, hábitos alimentares inadequados e altos níveis de estresse no ambiente de trabalho, que são condições frequentemente presentes nessa profissão. Os profissionais da área da saúde são expostos a condições de trabalho extremas, como elevada carga de trabalho, horários irregulares e turnos de longa duração; essas circunstâncias comprometem sua qualidade de vida e os predispõem a hábitos não saudáveis, como alimentação inadequada e ausência de prática de atividade física, o que aumenta o risco de sobrepeso e obesidade^(13,21)^.

Segundo evidências recentes, a alfabetização em saúde é classificada como essencial para o controle do sobrepeso/obesidade e conscientização necessária à superação da doença. A atuação do profissional de saúde na educação em saúde da população é de grande relevância^(25)^. Sendo assim, sobrepeso/obesidade entre os profissionais da saúde pode minar sua credibilidade diante dos pacientes, reduzindo a adesão às orientações fornecidas. Além disso, os profissionais de saúde obesos sentem-se menos confiantes para aconselhar os pacientes sobre alimentação saudável e sobre a importância da prática de exercícios físicos^(21)^.

No presente estudo, foi observada maior prevalência de sobrepeso/obesidade nos profissionais de saúde entre 30 e 39 anos. Esse resultado converge com aquele de um estudo de coorte de base populacional no Reino Unido, no qual se observou que a associação entre IMC e mortalidade foi mais forte na população com idade inferior a 50 anos^(26)^. Em uma pesquisa feita em Recife, capital do estado de Pernambuco, observou-se também uma associação estatisticamente significante entre o sobrepeso/obesidade e faixa etária, com uma maior prevalência em adultos de 30 a 39 anos, correspondendo a 27,5% dos classificados como obesos^(27)^. A diminuição nos níveis de atividade física e as alterações do organismo no processo de envelhecimento levam ao aumento no estoque de gordura corporal, excedendo os padrões aceitáveis de normalidade antropométrica^(27)^.

Nosso estudo mostrou que o sobrepeso/obesidade associou-se a alterações na pressão arterial e nos triglicérides. O acúmulo de gordura que ocorre na obesidade ocasiona a dislipidemia, a elevação dos níveis séricos de colesterol total (CT), bem como a fração de colesterol da lipoproteína de baixa densidade (LDL) e de triglicérides^(28)^. O sobrepeso/obesidade produz o aumento de adipocitocinas no organismo e, consequentemente, um estado inflamatório crônico, o que contribui para a manifestação de complicações metabólicas, como a hipertensão^(29)^. Quando o sobrepeso/obesidade e a hipertensão coexistem, ocorre um remodelamento cardíaco adverso, aumentando os riscos de insuficiência cardíaca^(30)^. Em um estudo realizado no nordeste do estado de Minas Gerais, com indivíduos entre 18 e 88 anos, foi encontrada uma associação entre os níveis elevados de triglicérides como fator de risco significativo para doenças cardiovasculares e, consequentemente, hipertensão arterial^(31)^. Já em uma investigação na comunidade de Kailuan, China, o nível mais alto do índice de triglicérides e a maior variabilidade do índice de triglicérides foram, cada um, independentemente associados a uma maior incidência de doenças cardiovasculares^(32)^.

No presente estudo, o sobrepeso/obesidade foi associado ao maior tempo de trabalho na área da saúde. Em estudo realizado com trabalhadores de saúde no Rio de Janeiro, observou-se que 53,5% tinham excesso de peso (36,7% com sobrepeso e 16,8% com obesidade). Esse resultado assemelha-se àquele do estudo na Malásia, mencionado anteriormente, em que 33,1% dos profissionais de saúde estavam acima do peso e 21,1% eram obesos. Os estudos mostraram que quanto mais tempo eles foram expostos às rotinas exaustivas inerentes às profissões da área da saúde, mais os hábitos de vida ficaram comprometidos, resultando em horários de trabalho irregulares e prolongados e má alimentação, predispondo-os à obesidade^(13,21)^.

### Limitações do estudo

É importante considerar as possíveis limitações deste estudo transversal, pois ele proporciona apenas uma visão instantânea da exposição e do desfecho, não sendo possível a avaliação temporal entre a causa e o efeito. Além disso, o viés de seleção e o viés de resposta podem ter interferido na coleta dos dados e, por sua vez, afetado a precisão dos resultados. Além disso, deve-se considerar que o equipamento utilizado para aferir a pressão arterial, embora validado, pode ser menos preciso do que dispositivos recomendados para estudos epidemiológicos.

### Contribuições para a área

Uma vez que a prevalência mundial de sobrepeso/obesidade entre os profissionais de saúde aumentou rápida e continuamente nas últimas décadas, urge criar estratégias, como ajustes na constante variação de turnos e na carga horária exaustiva, para garantir uma rotina mais saudável e melhorar a qualidade de vida desses trabalhadores. Portanto, este estudo é relevante por categorizar quais as consequências atuais e futuras para os profissionais de saúde. Os impactos físicos e psicológicos levam à redução na qualidade de vida e na capacidade de trabalho, o que aumenta a incidência de adoecimento.

## CONCLUSÕES

Conclui-se que a elevada prevalência de sobrepeso/obesidade entre profissionais de saúde está associada a características sociodemográficas, ocupacionais e condições de saúde, incluindo fatores como envelhecimento, duração da carreira e indicadores de saúde.

Os achados ressaltam a importância de implementar programas que incentivem a prática regular de exercícios físicos e a adoção de hábitos alimentares saudáveis, visando melhorar a qualidade de vida e prevenir doenças relacionadas ao sobrepeso/obesidade. Além disso, é essencial estabelecer redes de apoio que ofereçam suporte contínuo para facilitar mudanças comportamentais. Essas iniciativas devem ser complementadas por políticas públicas e ações específicas voltadas para a saúde ocupacional desses profissionais, a fim de promover um ambiente de trabalho mais saudável e equilibrado. Por fim, sugere-se a realização de pesquisas longitudinais com amostras em diferentes cenários hospitalares.

## Data Availability

Os dados de pesquisa só estão disponíveis mediante solicitação.
