# Supplementary material for: Erratum: Overweight/obesity among health professionals and associated factors
Source: Rev Bras Enferm. 2026 Aug 7;79:e2026e0220240254. doi: 10.1590/0034-7167.202679e0220240254 (PMC13450935; doi:10.1590/0034-7167.202679e0220240254)
Supplement: Related texts

# Correction: Errata: Sobrepeso/obesidade em profissionais de saúde e fatores associados

**DOI:** 10.1590/0034-7167.202679e0220240254pt

No artigo “Sobrepeso/obesidade em profissionais de saúde e fatores associados”, com número DOI: https://doi.org/10.1590/0034-7167-2024-0254pt, publicado no periódico Revista Brasileira de Enfermagem, 2025;78(6):e20240254:

Inclui-se:

**FOMENTO**

Fundação de Amparo à Pesquisa do Estado de Minas Gerais (Fapemig) - Processo APQ-04716-24, aprovado pelo Edital Nº 009/2024 - Fortalecimento e consolidação da pesquisa na UEMG E UNIMONTES.

Profa. Dra. Dulce Barbosa

*Editora-chefe*
